# Organic Fertilizer Substitution Modulates Soil Properties and Microbial Communities in a Vegetable–Earthworm Co-Cultivation System

**DOI:** 10.3390/microorganisms13122742

**Published:** 2025-12-01

**Authors:** Shumei Cai, Sixin Xu, Deshan Zhang, Yun Liang, Xianqing Zheng, Haitao Zhu

**Affiliations:** 1 Institute of Eco-Environment and Plant Protection, Shanghai Academy of Agricultural Sciences, Shanghai 201403, China; caishumei@saas.sh.cn (S.C.); xusixin@saas.sh.cn (S.X.); zds234@163.com (D.Z.); liangyun@saas.sh.cn (Y.L.); zxqfxf@163.com (X.Z.); 2 Key Laboratory of Low-Carbon Green Agriculture, Ministry of Agriculture and Rural Affairs, Shanghai 201403, China; 3 Shanghai Key Laboratory of Horticultural Technology, Shanghai 201403, China

**Keywords:** vegetable–earthworm co-culture, partial organic fertilization, soil biotillage, microbial diversity, microbial community stability, sustainable agriculture

## Abstract

In intensive vegetable production systems, long-term reliance on chemical fertilizers often leads to soil degradation and microbial imbalance, highlighting the need for sustainable biotillage strategies. In this study, a long-term field experiment examined how vegetable–earthworm co-cultivation (VE) combined with different fertilization regimes affects vegetable yield, soil physicochemical properties, and microbial communities. VE significantly improved vegetable yield, with full chemical fertilization (VE_IF100) and a 30% reduction in chemical fertilizer supplemented with organic fertilizer (VE_IF70) increasing yields by 30.86% and 26.02%, respectively, relative to full fertilization without earthworms (CK_IF100). VE also moderated soil pH toward neutrality. VE_IF100 decreased the soil C/N ratio, whereas VE_IF70 increased it and enhanced available hydrolyzable nitrogen, indicating a more balanced nutrient transformation. Microbial analysis revealed that VE_IF100 reduced bacterial abundance while strongly increasing fungal abundance, decreasing the bacteria-to-fungi ratio from 3.51 to 0.53. In contrast, VE_IF70 restored the bacteria-to-fungi ratio to 1.65 and increased fungal diversity, with the Shannon and Chao1 indices exceeding those in VE_IF100. Bacterial genera associated with nutrient cycling and plant growth promotion (e.g., *Brevundimonas*, *Anaeromyxobacter*) were enriched under VE_IF70, while fungal taxa with antagonistic and biocontrol potential (e.g., *Chaetomium*, *Arthrobotrys*) also increased. Redundancy analysis identified the soil C/N ratio (ranging from 5.94 to 8.60 across treatments) as a key driver of both bacterial and fungal community structures, whereas pH exerted a stronger influence on fungi. Random forest analysis indicated that the annual total vegetable yield was primarily driven by fertilization and available phosphorus in VE systems, whereas pH and bacterial abundance were the main drivers in CK systems. Overall, earthworm inoculation combined with partial organic fertilizer substitution improved soil conditions, reshaped microbial communities, and maintained high yield, demonstrating a practical strategy for sustainable vegetable production.

## 1. Introduction

The rapid intensification of vegetable production has considerably boosted crop yields [1], yet it has simultaneously contributed to environmental pollution and heightened risks to human health [2,3]. Prolonged and excessive reliance on chemical fertilizers has been shown to deplete soil organic matter, aggravate compaction and acidification, and disrupt the diversity and functional stability of rhizosphere microbial communities [4,5]. Such alterations ultimately threaten the long-term productivity and ecological resilience of agroecosystems [6]. Therefore, reducing chemical fertilizer inputs while supplementing with organic amendments has emerged as a crucial strategy for promoting sustainable agricultural transformation [7]. Increasing evidence indicates that partial substitution of chemical fertilizers with organic sources can enhance soil organic matter content and nutrient retention [8], thereby fostering the recovery and functional stability of soil microbial communities [9].

Earthworms, regarded as ecosystem engineers, offer unique advantages in improving soil physicochemical properties and nutrient cycling [10,11]. Both global and regional field studies have shown that earthworm activity promotes soil aggregate formation, accelerates organic matter decomposition, and enhances nutrient availability, ultimately contributing to yield improvements in various cropping systems [11,12]. These functions make earthworm-based biotillage a promising integrated strategy for sustainable farmland management.

Earthworms interact intricately with soil microorganisms through their ingestion, digestion, excretion, and burrowing activities [13,14]. On one hand, these processes directly modify the composition of microbial communities and enrich specific functional taxa within their surrounding soil environment [15]. On the other hand, earthworm-mediated alterations in soil structure, aeration, and nutrient distribution indirectly reshape the spatial and nutritional niches of microorganisms, thereby influencing community functionality and modulating potential pathogen risks [16]. Earthworms can both suppress certain pathogens and stimulate beneficial microorganisms, but may also enhance fungal proliferation or alter pathogen–antagonist relationships, depending on earthworm species, soil type, environmental conditions, and fertilization regime [17]. The effects of organic fertilizer substitution and earthworm inoculation on soil quality and microbial functions have been widely documented, mainly focusing on the application of compost or vermicompost as soil amendments and the role of earthworm inoculation in regulating microbial processes, including carbon and nitrogen cycling enzyme activities. Organic substitution stabilizes soil microbial networks and enhances the abundance of functional genes; however, its interaction with earthworm activity remains uncertain, particularly regarding whether organic inputs amplify or mitigate the microbial proliferation associated with earthworms [18,19].

This study aims to contribute to ecological intensification by examining how vegetable–earthworm co-cultivation (VE), in combination with chemical-to-organic fertilizer substitution, can maintain crop yields while improving soil health and restructuring microbial networks toward more resilient and low-carbon systems. Despite increasing interest in both practices, current understanding remains limited by several methodological constraints, including the predominance of short-term or laboratory experiments, inconsistencies in earthworm species and inoculation densities, large variability in soil and climatic conditions, and discrepancies between predicted and observed microbial functions. Such limitations have restricted the development of cross-regional insights and standardized management strategies.

To address these challenges, we conducted a long-term field experiment comparing conventional monoculture vegetable systems with VE systems under four fertilization regimes: full chemical fertilizer, 30% chemical fertilizer reduction with organic substitution, full organic fertilizer, and no fertilization. The study assessed treatment effects on vegetable yield, soil physicochemical properties, and the abundance, diversity, composition, and functional attributes of soil microbial communities. We hypothesized that (1) organic fertilizer substitution within VE systems would enhance soil nutrient availability and sustain crop productivity; (2) key soil environmental factors would act as hierarchical drivers of the coupled microbe–yield response, modulated by earthworm activity; and (3) partial substitution of chemical fertilizer with organic inputs would promote microbial community stabilization and disease risk mitigation through community restructuring.

## 2. Materials and Methods

### 2.1. Experimental Design

The field experiment was conducted at the Zhuanghang Comprehensive Experimental Station of the Shanghai Academy of Agricultural Sciences (120.23° E, 30.54° N). The vegetable–earthworm co-culture plots were established in April 2015. The soil at the experimental site is classified as a *Fluvisol* according to the World Reference Base for Soil Resources (WRB) [20], with a profile comprising an Ap horizon (0–20 cm) overlying a weakly structured alluvial subsoil (20–60 cm). The mean particle size distribution at the sampling sites comprised 39.3% sand (2–0.05 mm), 45.7% silt (0.05–0.002 mm), and 15.0% clay (<0.002 mm). The basic physicochemical properties of the 0–20 cm soil layer in March 2015, prior to the start of the experiment, were measured to characterize the baseline soil conditions: total organic carbon (TOC) 7.42 g kg^−1^; bulk density 1.15 g cm^−3^; total soil porosity 56.6%; pH 6.95; alkali-hydrolysable nitrogen (AHN) 80.2 mg kg^−1^; available phosphorus (AP) 62.5 mg kg^−1^; and available potassium (AK) 198.3 mg kg^−1^.

The experiment was established to assess the effects of vegetable–earthworm co-cultivation (VE) under different fertilization regimes. Six treatments were established to evaluate the effects of fertilization regimes and earthworm co-cultivation. Four treatments included earthworms: (1) VE_IF100, receiving the full rate of inorganic fertilizer; (2) VE_IF70, with a 30% reduction in inorganic nitrogen fertilizer compensated by organic fertilizer; (3) VE_OF100, supplied exclusively with organic fertilizer; and (4) VE_0, without fertilization. To distinguish the effects of earthworm activity, two additional non-earthworm controls were included: (5) CK_IF100, full-rate inorganic fertilizer without earthworms; and (6) CK_0, unfertilized control without earthworms. Calcium nitrate tetrahydrate (N% = 11.86%), monopotassium phosphate (P% = 22.79%, K% = 28.68%) and potassium sulphate (K% = 44.83%) were applied as the experimental N, P and K fertilizer. The commercial composted manure–based organic fertilizer, supplied by Shanghai Huita Industrial Co., Ltd. (Shanghai, China), contained 408.2 g kg^−1^ organic matter, 17.3 g kg^−1^ N, 14.7 g kg^−1^ P_2_O_5_, and 13.6 g kg^−1^ K_2_O.

The crop rotation scheme involved alternating *Brassica oleracea* L. with *Colocasia esculenta* L. Except for the unfertilized treatments (VE_0 and CK_0), all other treatments received the same total nutrient inputs, with *B. oleracea* supplied with 403.5, 118.5, and 285.0 kg ha^−1^ of N, P_2_O_5_, and K_2_O, respectively, and *C. esculenta* supplied with 441.0, 190.5, and 552.0 kg ha^−1^, respectively. Earthworms (*Pheretima guillelmi*), a dominant local native species widely used to improve soil quality, were inoculated at a rate of 1500 kg ha^−1^. Each plot measured 7.2 m × 5.2 m (37.44 m^2^) and was separated from adjacent plots by 0.5 m buffer ridges. The experiment followed a randomized complete block design with three replicates per treatment. Weeding was performed manually, and other field management practices were consistent across treatments.

### 2.2. Crop Harvest and Soil Sampling

*C. esculenta* and *B. oleracea* were harvested on 14 October 2019, and 9 March 2020, respectively. Crop yield was determined by weighing the total fresh biomass harvested from each plot. Following the harvest of *B. oleracea*, composite topsoil samples were collected using an S-shaped five-point sampling strategy (Figure S1). Each composite sample was homogenized, collected and sieved through a 4 mm mesh to remove large stones, gravel, plant roots and other non-soil materials. Prior to mixing, the soil samples were divided into two groups: one was air dried for the determination of basic physicochemical properties, and the other was stored at −80 °C in preparation for high-throughput gene sequencing and qPCR analysis.

### 2.3. Determination of Soil Physicochemical Parameters

Soil physicochemical parameters, including TOC, TN, AHN, AP, AK, total dissolved solids (TDS), and pH, were assessed using the methods described by Lu [21]. TOC was determined using the potassium dichromate oxidation–spectrophotometric method, and total nitrogen (TN) was measured by the Kjeldahl digestion method. AHN was measured with the alkaline hydrolysis diffusion method. AP was extracted with 0.5 mol L^−1^ NaHCO_3_ and determined by the molybdenum–antimony colorimetric method. AK was extracted with 1 mol L^−1^ ammonium acetate and measured by flame photometry. TDS was determined using a gravimetric method, and soil pH was measured in CO_2_–free distilled water (soil:water = 1:2.5) using a pH meter (FE28, Mettler Toledo, Giessen, Germany). Soil bulk density (BD) and total soil porosity (TSP) were determined by ring knife method [22]. Soil mechanical composition was determined by Mastersizer 3000 laser particle size analyzer (Malvern Panalytical, Malvern, UK) [23].

### 2.4. Soil DNA Extraction and PCR Amplification

Total DNA was extracted from 0.5 g of the −80 °C preserved soil subsamples using the MoBio PowerSoil^®^ DNA Isolation Kit (MoBio Laboratories, Carlsbad, CA, USA), and DNA concentration and purity were determined with a NanoDrop 2000 spectrophotometer (Thermo Scientific, Waltham, MA, USA). One DNA extraction was conducted for each composite soil sample. Extracted DNA was stored at −20 °C for downstream analyses. The V3–V4 region of the bacterial 16S rRNA gene was amplified using primers 338F (5′-ACTCCTACGGGAGGCAGCAG-3′) and 806R (5′-GGACTACHVGGGTWTCTAAT-3′). The fungal ITS region was amplified using primers ITS1F (5′-CTTGGTCATTTAGAGGAAGTAA-3′) and ITS2R (5′-GCTGCGTTCTTCATCGATGC-3′). PCR reactions were performed using FastPfu polymerase (Takara, Dalian, China) following the manufacturer’s instructions, with cycling conditions according to Cai et al. [24]. Amplicons were verified on 1.7% agarose gels prior to sequencing.

### 2.5. Illumina MiSeq Sequencing and Data Processing

Verified PCR products were sequenced on the Illumina MiSeq PE250 platform (Majorbio Bio-Pharm Technology Co., Ltd., Shanghai, China). Raw FASTQ files were quality filtered using Trimmomatic (v0.33) and merged with FLASH (v1.2.7). Operational taxonomic units (OTUs) were clustered at 97% similarity using the UPARSE pipeline (v7.1). Taxonomic assignments were performed against the RDP (for bacteria) and UNITE (for fungi) databases. After quality control, a total of 934,548 bacterial 16S rRNA sequences (average length 418.9 bp) and 1,076,667 fungal ITS sequences (average length 243.3 bp) were obtained across the six treatments (*n* = 18). The sequencing depth was sufficient for downstream analysis, with an average of 51,919 ± 6371 reads per sample for bacteria (range: 45,564–63,265) and 59,815 ± 10,206 reads per sample for fungi (range: 48,159–70,051). The sequence numbers for each individual sample are provided in Supplementary Table S1. Sequence data have been deposited in the NCBI Sequence Read Archive (SRA) under accession number SRP415876.

### 2.6. Soil Quantitative PCR

Bacterial 16S rRNA and fungal ITS gene copy numbers were quantified using an ABI Prism 7300 Real-Time PCR System (Applied Biosystems, Carlsbad, CA, USA). Standard curves were generated from plasmid clones containing the respective 16S or ITS gene inserts through 10-fold serial dilutions.

For 16S rRNA, reactions were performed in 20 μL volumes containing 10 μL of 2× Taq Plus Master Mix (Takara, Dalian, China), 0.8 μL of each primer (5 μM), 7.4 μL of sterilized water, and 1 μL of extracted soil DNA. Thermal cycling included an initial denaturation at 95 °C for 5 min, followed by 40 cycles of 95 °C for 30 s (denaturation), 58 °C for 30 s (annealing and extension), and a final step at 72 °C for 1 min. For ITS, qPCR was performed in a 20 μL reaction mixture containing 10 μL of SYBR Premix Ex Taq™ (Takara, Dalian, China), 1.0 μL of each primer (10 μM), 7.0 μL of sterilized water, and 1 μL of extracted soil DNA. Thermal cycling included an initial denaturation at 95 °C for 30 s, followed by 40 cycles of 95 °C for 5 s (denaturation) and 60 °C for 30 s (annealing and extension), with a final cooling step at 50 °C for 30 s.

Specificity of qPCR products was confirmed by melting curve analysis and gel electrophoresis. Negative controls were included in all assays. Gene copy numbers were calculated based on standard curves and expressed as copies per gram of soil. Although the standard curves account for qPCR efficiency, absolute abundances may still be influenced by DNA extraction efficiency and other experimental factors, and thus should be interpreted as approximate estimates.

### 2.7. Statistical Analyses

Significant differences in soil physicochemical properties among the six treatments were evaluated using one-way ANOVA followed by Tukey’s HSD test (SPSS 19.0, IBM, Armonk, NY, USA). As the treatments did not constitute a fully crossed factorial design, a conventional two-way ANOVA for estimating main effects and interactions was not feasible; consequently, the six treatments were considered as independent groups in the one-way ANOVA. Alpha-diversity indices (Chao1 and Shannon) for both bacteria and fungi were calculated using Mothur (v1.45.3) to assess microbial richness and diversity, respectively [25,26]. Taxa contributing significantly to differences in bacterial and fungal community composition were identified by linear discriminant analysis effect size (LEfSe) via the Galaxy platform (http://huttenhower.sph.harvard.edu/galaxy/, accessed on 15 October 2020) [27]. Redundancy analysis (RDA) was performed using the vegan package in R (v4.3.2) to explore relationships between microbial community composition and environmental variables [28], while Pearson correlation analysis was applied to assess associations between microbial α-diversity and soil physicochemical properties. To determine the relative importance of soil and microbial factors in explaining yield variations between the two cultivation systems, a random forest model was fitted in R with 1000 trees (ntree = 1000) and default mtry. Variable importance was evaluated using the increase in node purity (IncNodePurity), and model performance was assessed using *R*^2^ [29].

## 3. Results

### 3.1. Vegetable Yield

**Figure 1:**
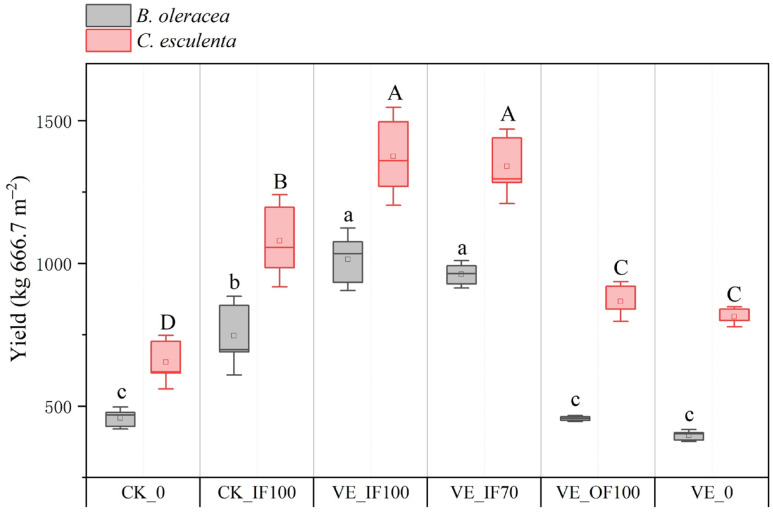
Effects of fertilization treatments on the annual total yield of vegetables (*B. oleracea* and *C. esculenta*) under vegetable–earthworm co–culture and vegetable–monoculture. Lowercase letters indicate significant differences (*p* < 0.05) among the yields of *B. oleracea*, while uppercase letters indicate significant differences (*p* < 0.05) among the yields of *C. esculenta*.

Earthworm inoculation markedly increased vegetable yield. The annual total yield of VE_IF100 and VE_IF70 was 30.86% and 26.02% greater than that of CK_IF100, respectively. In the unfertilized treatments, VE_0 increased vegetable yield by 8.75% compared with CK_0. Both VE_IF100 and VE_IF70 exhibited significantly higher total yields than VE_OF100 and VE_0, while no significant difference was observed between VE_IF100 and VE_IF70 (Figure 1). Compared with CK_IF100, the yields of *B. oleracea* and *C. esculenta* under VE_IF100 treatment increased by 35.84% and 27.42%, respectively, with the former showing a stronger yield-promoting effect.

### 3.2. Soil Physicochemical Properties

**Figure 2:**
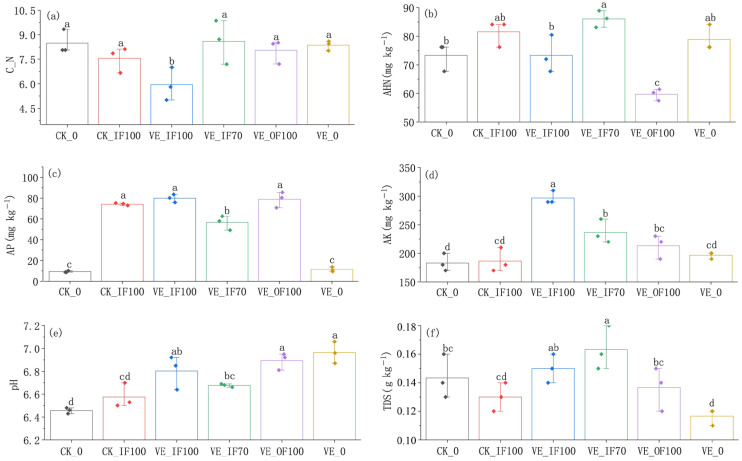
Effects of fertilization treatments on soil properties in vegetable–earthworm co-culture and monoculture systems. (**a**) Soil carbon to nitrogen ratio (C:N); (**b**) Alkali-hydrolysable nitrogen (AHN, mg kg^−1^); (**c**) Available phosphorus (AP, mg kg^−1^); (**d**) Available potassium (AK, mg kg^−1^); (**e**) Soil pH; (**f**) Total dissolved solids (TDS, g kg^−1^). Lowercase letters indicate significant differences (*p* < 0.05) among the treatments.

Earthworm inoculation elevated soil pH (Figure 2e), shifting it toward near-neutral conditions (6.68–6.89). Compared with CK_IF100, VE_IF100 decreased the soil C/N ratio but increased the contents of available potassium (AK) and total dissolved solids (TDS) (Figure 2a,d,f). In comparison with VE_IF100, VE_IF70 exhibited higher C/N and available hydrolyzable nitrogen (AHN) but lower available phosphorus (AP) and AK contents (Figure 2a–d). The VE_0 treatment significantly increased soil pH and reduced TDS compared with CK_0 (Figure 2e,f).

### 3.3. Soil Bacterial and Fungal Abundance and Diversity Indices

Compared with CK_IF100, the bacterial abundance in VE_IF100 decreased by 48.25%, while fungal abundance increased by 241.52%, resulting in a decline of the bacterial-to-fungal (B/F) ratio from 3.51 to 0.53 (Table 1). Relative to VE_IF100, VE_IF70 exhibited a 30.55% increase in bacterial abundance and a 57.71% reduction in fungal abundance.

Meanwhile, the Shannon and Chao1 diversity indices of fungi increased by 24.64% and 19.95%, respectively, and the bacterial-to-fungal ratio rose to 1.65. In VE_OF100, both bacterial and fungal abundances decreased by 40.19% and 61.82%, respectively. Compared with CK_0, VE_0 increased fungal abundance by 62.62%, whereas the Shannon and Chao1 indices of fungi decreased by 26.94% and 29.07%, respectively.

### 3.4. Soil Bacterial Community Composition and Functions

**Figure 3:**
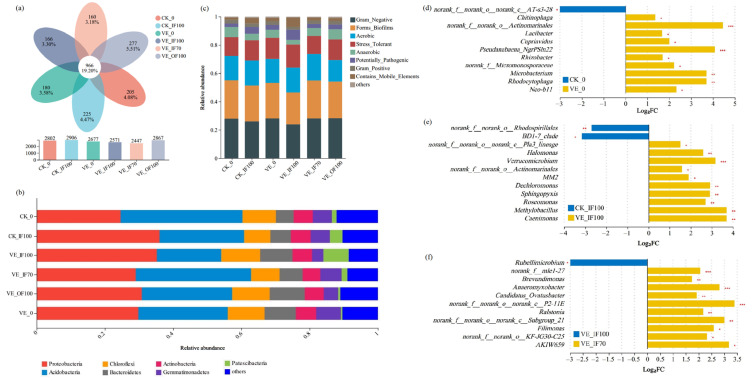
Bacterial community composition and predicted functional differences under earthworm inoculation and fertilization treatments. (**a**) Venn diagram of OTU-level composition (*y*-axis: total OTUs per treatment); (**b**) Phylum-level composition, with taxa < 0.05 merged as “others”; (**c**) BugBase-predicted phenotypes, with groups < 0.01 merged as “others”; (**d**–**f**) Genus-level differential taxa (top 10 genera), showing log2 fold changes between treatments and controls. Taxa enriched in treatments are indicated in treatment colors, and those reduced are indicated in control colors. Significance was assessed using two-tailed Fisher’s exact test. Panels d, e, and f correspond to CK_0 vs. VE_0, CK_IF100 vs. VE_IF100, and VE_IF100 vs. VE_IF70, respectively. * 0.01 < *p* ≤ 0.05, ** 0.001 < *p* ≤ 0.01, *** *p* ≤ 0.001.

A total of 966 bacterial OTUs (19.20%) were shared among all treatments. CK_IF100 had the highest OTU richness (2906 OTUs), whereas VE_IF70 had the lowest (2447 OTUs) (Figure 3a). Although richness declined, VE treatments substantially reshaped the bacterial community relative to CK. At the phylum level, VE_IF100 showed substantial increases in Chloroflexi (by 48.37%) and Patescibacteria (by 100.54%) but a decrease in Acidobacteria (by 31.39%) relative to CK_IF100 (Figure 3b), while VE_IF70 was enriched in Acidobacteria (33.8%) and Gemmatimonadetes (6.2%), indicating a distinct pattern of community assembly. At the genus level, VE treatments generally enriched *Flavobacterium*, *unclassified_o__Ignavibacteriales*, *Pseudoxanthomonas*, *Dyadobacter*, *Lacunisphaera*, *Actinophytocola*, *Sphingopyxis*, and *Azospira* compared with CK (Figure S2a). Specifically, VE_0 was characterized by higher abundances of *Actinomarinales*, *Cupriavidus*, and *Microbacterium* (Figure 3d); The VE_IF100 treatment was dominated by genera including *Methylobacillus* and *Dechloromonas* (Figure 3e), whereas *Brevundimonas* and *Anaeromyxobacter* were among the predominant taxa in VE_IF70 (Figure 3f).

BugBase functional prediction further indicated that VE_IF70 contained a higher proportion of Gram-negative bacteria (increased by 17.27%), a shift that occurred alongside the enrichment of Acidobacteria and Gemmatimonadetes (Figure 3b) and may reflect improved nutrient-cycling conditions favoring beneficial functional groups, together with a markedly lower abundance of potential pathogens (reduced by 60.25%) compared with CK_IF100. In contrast, VE_IF100 exhibited a 54.41% increase in potential pathogenic bacteria (Figure 3c).

### 3.5. Soil Fungal Community Composition and Functions

**Figure 4:**
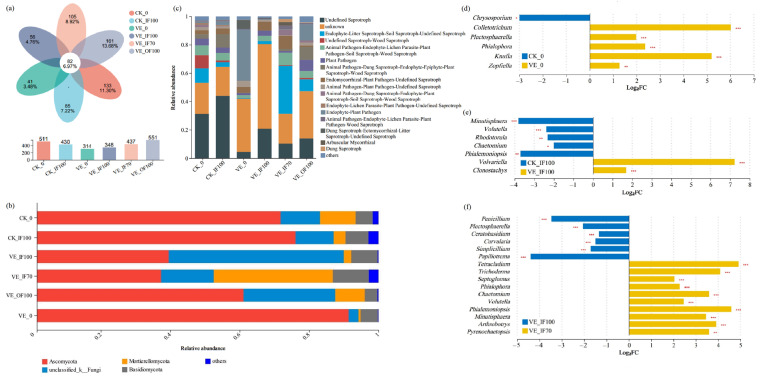
Fungal community composition and predicted functional differences under earthworm inoculation and fertilization treatments. (**a**) Venn diagram of OTU-level composition (*y*-axis: total OTUs per treatment); (**b**) Phylum-level composition, with taxa < 0.05 merged as “others”; (**c**) FUNGuild-based functional prediction, with groups < 0.01 merged as “others”; (**d**–**f**) Genus-level differential taxa (top 10 genera), showing log2 fold changes between treatments and controls. Taxa enriched in treatments are indicated in treatment colors, and those reduced are indicated in control colors. Significance was assessed using two-tailed Fisher’s exact test. Panels d, e, and f correspond to CK_0 vs. VE_0, CK_IF100 vs. VE_IF100, and VE_IF100 vs. VE_IF70, respectively. * 0.01 < *p* ≤ 0.05, ** 0.001 < *p* ≤ 0.01, *** *p* ≤ 0.001.

A total of 82 fungal OTUs (6.97%) were shared across all treatments. VE_OF100 exhibited the highest OTU richness (551 OTUs), while VE_IF100 and CK_IF100 showed reductions of 36.84% and 21.96%, respectively. Notably, OTU richness in VE_IF70 increased by 25.57% relative to VE_IF100 (Figure 4a). At the phylum level, the relative abundance of Ascomycota in VE_IF100 was 48.0% lower than in CK_IF100, whereas Mortierellomycota accounted for 34.3% in VE_IF70 (Figure 4b). At the genus level, VE were significantly enriched in *unclassified_k__Fungi*, *Olpidium*, *Clonostachys*, *Entoloma*, and *unclassified_c__Agaricomycetes* compared with CK (Figure S2b). Specifically, VE_IF100 was enriched in *Volvariella* and *Clonostachy* (Figure 4e), while VE_IF70 was enriched in *Chaetomium*, *Arthrobotrys*, and *Phialemoniopsis* (Figure 4f), suggesting a more functionally diverse fungal community enriched in beneficial taxa.

FUNGuild functional prediction further indicated clear divergence in fungal ecological roles among treatments. CK_IF100 was dominated by undefined saprotrophs (43.91%), whereas VE_IF70 was characterized by greater proportions of multifunctional trophic modes (33.76%) and increased abundances of arbuscular mycorrhizal fungi (2.60%). VE_IF70 also showed an increase in endomycorrhizal–plant pathogen–undefined saprotroph (9.28%), a group of genera containing species with different trophic modes (i.e., fungi that function as endomycorrhizal symbionts, plant pathogens, or saprotrophs). In contrast, plant pathogenic guilds, which may have pathogenic effects on plants, were most abundant in VE_OF100 (19.31%) and VE_0 (37.80%).

### 3.6. Environmental Drivers of Soil Microbial Community Shifts and Yield Attribution

**Figure 5:**
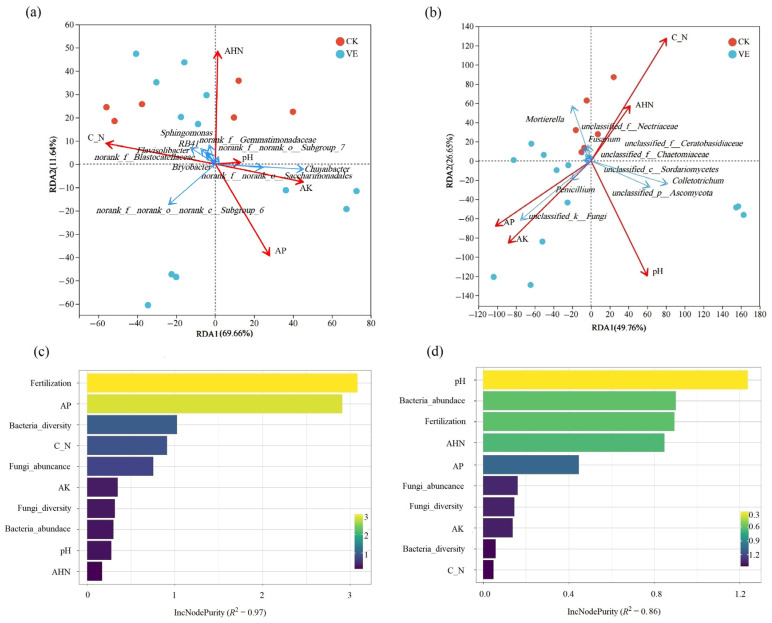
Microbial–environment interactions and yield-driving factors under earthworm inoculation and fertilization treatments. (**a**) RDA of bacterial and fungal communities at the phylum level in relation to environmental factors; (**b**) RDA of fungal communities at the phylum level in relation to environmental factors; (**c**) Random forest ranking of variables driving vegetable yield in the VE system; (**d**) Random forest ranking of variables driving vegetable yield in the CK system. Alpha diversity of bacterial and fungal communities was assessed using the Shannon index, which captures both richness and evenness.

RDA revealed that soil C/N ratio exhibited the strongest influence on bacterial community composition (R^2^ = 0.9873, *p* = 0.0097), followed by AHN (Figure 5a, Table S2). The dominant genera such as *Sphingomonas*, *Flavisolibacter* and *Bryobacter* were positively correlated with both factors. For fungi, C/N ratio (R^2^ = 0.9055, *p* = 0.0639) and pH (R^2^ = 0.7114, *p* = 0.0722) were the dominant determinants (Figure 5b). Notably, dominant taxa such as *Mortierella* and *Fusarium* were negatively correlated with pH, whereas *Colletotrichum* and *unclassified Ascomycota* showed the opposite pattern.

Random forest analysis further identified distinct yield-driving mechanisms under different systems (Figure 5c,d). In the earthworm-inoculated VE system, fertilization and AP were the primary yield predictors, followed by bacterial diversity, whereas pH and bacterial abundance dominated in the CK system. Overall, the VE system exhibited stronger coupling between nutrient availability, microbial diversity, and yield, underscoring the enhanced regulatory role of earthworm inoculation in promoting crop productivity.

## 4. Discussion

### 4.1. Earthworm-Mediated Enhancement of Vegetable Yield

Earthworm inoculation markedly enhanced vegetable yields, with VE_IF100 and VE_IF70 exhibiting 30.86% and 26.02% higher productivity than CK_IF100, respectively. This improvement primarily resulted from the multifaceted functions of earthworms, whose continuous burrowing and casting activities enhanced soil aeration, aggregate stability, and root–soil contact, thereby improving nutrient availability and uptake efficiency in the rhizosphere [30,31]. By biologically mixing organic residues with mineral particles, earthworms further accelerated the decomposition and mineralization of labile organic matter, promoting gradual nutrient release [32,33]. Importantly, their burrowing, ingestion, and casting behaviors help redistribute nutrients more evenly through the soil, thus avoiding localized accumulation and promoting more homogeneous nutrient availability for plant uptake [11].

Earthworm presence also helped buffer soil pH and improve nutrient availability, consistent with their well-documented role in enhancing soil fertility through accelerated organic matter turnover and nitrogen cycling [34]. The nutrient transformation pattern, however, varied among fertilization regimes. VE_IF100 decreased the soil C/N ratio relative to CK_IF100, indicating accelerated mineralization driven by intense earthworm activity, whereas VE_IF70 increased the C/N ratio and significantly elevated AMN and AHN levels in the soil, suggesting a more balanced and efficient nutrient conversion process that sustained nitrogen supply for crop uptake.

In addition, our study confirmed that available phosphorus and bacterial Shannon diversity were the major predictors of yield variation, emphasizing the pivotal role of earthworms as biological integrators that connect soil physicochemical conditions, microbial diversity, and crop productivity. Taken together, these results confirm our first hypothesis that earthworms can maintain or even enhance vegetable yields, providing a mechanistic basis for sustainable intensification of vegetable production systems.

### 4.2. Synergistic Effects of Earthworms and Partial Organic Substitution on Microbial Community Structure

The interaction between earthworms and organic fertilizer substitution profoundly reshaped the rhizosphere microbial community in both composition and function [35]. Under full chemical fertilization, earthworm activity (VE_IF100) markedly reduced bacterial abundance while promoting fungal proliferation, shifting the bacterial-to-fungal (B/F) ratio from 3.51 to 0.53. Although fungal enrichment accelerates organic matter decomposition, it may also increase pathogen pressure and weaken microbial network stability. In contrast, partial organic substitution (VE_IF70) restored the B/F balance (1.65) and increased fungal diversity, consistent with the view that the bacterial-to-fungal ratio is a useful indicator of soil microbial equilibrium and stress responses [36]. This suggests that earthworm inoculation combined with moderate organic inputs promotes a more balanced and resilient microbial assemblage [37,38]. Further, VE_IF70 was enriched in bacterial taxa associated with nutrient cycling and plant growth promotion, such as *Brevundimonas* and *Anaeromyxobacter* at the genus level (Figure 3f) [39,40], while fungal taxa with well-documented antagonistic or biocontrol activities, including *Chaetomium* and *Arthrobotrys*, were also more abundant (Figure 4f) [41,42]. These compositional changes indicate that the interaction between earthworms and organic amendments selectively promoted functional groups beneficial to nutrient transformation and pathogen suppression [43]. This supports our second hypothesis that soil environmental factors, particularly the C/N ratio and available phosphorus, act as key mediators linking earthworm-driven nutrient dynamics with microbial restructuring and yield improvement.

Functional predictions further reinforced these findings. BugBase analysis revealed that VE_IF70 harbored a higher proportion of Gram-negative bacteria with versatile metabolisms and a markedly lower abundance of potential pathogens compared with VE_IF100 and CK_IF100. Meanwhile, FUNGuild analysis identified enrichment of Endophyte–Litter Saprotroph–Soil Saprotroph guilds, along with an increase in arbuscular mycorrhizal fungi and genera containing both symbiotic and pathogenic species, indicating enhanced niche differentiation and functional redundancy [44]. Such functional diversification enhances ecosystem stability and resistance to disturbance. By moderating nutrient release and creating microsites [45], earthworms likely buffered the microbial imbalance induced by excessive fertilization, maintaining relatively high bacterial and fungal diversity under VE_IF70. This diverse and functionally balanced community structure underlies system resilience and reduced pathogen incidence [46].

Taken together, these results confirm our third hypothesis that partial organic substitution under earthworm helps stabilize microbial communities and reduce pathogen risk, achieving ecological balance while maintaining crop productivity [30,47,48].

### 4.3. Fertilization Thresholds and Bidirectional Effects of Earthworm–Organic Interactions

Fertilization exerted a notable suppressive effect on bacterial abundance and diversity in vegetable fields, with a more pronounced reduction in the CK system (Figure S3b). This pattern highlights the context-dependent role of earthworms: under moderate nutrient inputs, earthworms enhance yield by improving nutrient cycling and stabilizing microbial communities, whereas under high nutrient loads, they may inadvertently promote pathogen proliferation, reflecting a bidirectional influence on soil microbial dynamics [49,50].

In VE systems, fertilization stimulated fungal abundance while reducing fungal diversity (Figure S3a). Under VE_IF100, fungal overproliferation occurred together with an enrichment of potential pathogens, such as *Ceratobasidium* and *Curvularia* (Figure 4f), indicating increased pathogen pressure. Although no visible disease incidence was detected at the early stage of this long-term experimental field, the rise in pathogen-associated taxa and the shift toward pathogenic fungal functions point to an elevated potential risk of soilborne diseases under this treatment. By contrast, VE_IF70 maintained high vegetable yield while optimizing microbial composition and functional potential. Functional shifts included enrichment of bacterial taxa involved in nutrient cycling and plant growth promotion, increased abundance of fungal antagonists and saprotrophs, and reduced prevalence of potential pathogenic fungi. These patterns indicate a fertilization threshold effect, whereby partial chemical replacement with organic amendments in conjunction with earthworm inoculation sustains productivity while preserving microbial balance [51].

Overall, these findings demonstrate that a management strategy integrating 70% chemical fertilizer, partial organic substitution, and earthworm inoculation (VE_IF70) can maintain crop productivity while stabilizing microbial communities, with yields 26.02% higher than those of CK_IF100 and comparable to those of VE_IF100. However, limitations include the single cropping system, reliance on community-level functional predictions, and indirect assessment of pathogen risks. To enhance generalizability, future studies should employ multi-site, long-term field experiments across contrasting soil types (e.g., clayey, loamy, sandy soils), combined with metagenomic and metatranscriptomic analyses. Direct pathogen monitoring is also recommended to elucidate interactive effects on soil health and crop performance.

## 5. Conclusions

The vegetable–earthworm co-cultivation (VE) system significantly enhanced cauliflower yield, even without additional chemical fertilizer input. In addition, VE moderated soil pH toward neutrality. Partial substitution of chemical fertilizer with 30% organic fertilizer (VE_IF70) further optimized soil nutrient status by increasing the C/N ratio and enhancing available hydrolyzable nitrogen (AHN), indicating more balanced nutrient transformation processes. VE_IF70 promoted bacterial proliferation while enhancing fungal diversity, as reflected by higher Shannon and Chao1 indices. It also restored the bacteria-to-fungi ratio from 0.53 (under VE_IF100) to 1.65, suggesting a more stable microbial equilibrium in the rhizosphere. Bacterial genera associated with nutrient cycling and plant growth promotion, such as Brevundimonas and Anaeromyxobacter, were selectively enriched, whereas fungal taxa with known antagonistic or biocontrol potential, including Chaetomium and Arthrobotrys, increased in relative abundance. The soil C/N ratio emerged as a principal environmental driver shaping both bacterial and fungal community structures, while pH exerted a stronger influence on fungi. By regulating the C/N ratio, VE_IF70 activated a cascade linking microbial functional restructuring with efficient nitrogen transformation and yield enhancement.

Collectively, these findings demonstrate that earthworm inoculation coupled with partial organic fertilizer substitution can sustain high productivity, improve soil nutrient balance, and stabilize microbial community composition. This integrated biotillage approach provides a practical framework for targeted microbial management in intensively cultivated or degraded vegetable fields, thereby advancing sustainable and resilient agricultural systems.

## Figures and Tables

**Table 1:** Effects of fertilization treatments on the abundance and diversity indices of bacterial and fungal communities in vegetable field soil under vegetable–earthworm co–culture and vegetable–monoculture.

Treatments	16S			ITS			B:F
	Quantity	Shannon	Chao1	Quantity	Shannon	Chao1	
	(10^9^ Copies/g)			(10^7^ Copies/g)			
**CK_0**	4.77 ± 0.18 b	**6.52 ± 0.03** a	3374.43 ± 68.20 b	2.06 ± 0.03 d	4.12 ± 0.05 ab	**562.66 ± 19.07** a	2.32 ± 0.10 b
**CK_IF100**	6.01 ± 0.34 a	**6.66 ± 0.04** a	**3706.73 ± 115.01** a	1.71 ± 0.07 e	**4.46 ± 0.04** a	**574.99 ± 22.85** a	3.51 ± 0.06 a
**VE_IF100**	3.11 ± 0.10 d	**6.53 ± 0.11** a	3355.27 ± 54.00 b	5.84 ± 0.02 a	2.76 ± 0.19 d	402.07 ± 24.65 c	0.53 ± 0.05 d
**VE_IF70**	4.07 ± 0.30 c	6.27 ± 0.19 ab	3287.77 ± 106.70 b	2.47 ± 0.05 c	3.44 ± 0.19 c	482.29 ± 31.56 b	1.65 ± 0.20 c
**VE_OF100**	1.85 ± 0.08 e	**6.42 ± 0.24** a	**3723.67 ± 90.03** a	2.23 ± 0.05 cd	3.93 ± 0.05 b	477.52 ± 11.87 b	0.83 ± 0.15 d
**VE_0**	4.84 ± 0.14 b	5.85 ± 0.21 b	3517.10 ± 99.12 ab	3.35 ± 0.17 b	3.01 ± 0.19 cd	399.12 ± 25.68 c	1.44 ± 0.02 c

Values in bold represent the statistically highest group for Shannon or Chao1 diversity based on one-way ANOVA with Tukey’s post hoc test (*p* < 0.05). Different letters indicate significant differences among treatments.

## Data Availability

The original contributions presented in this study are included in the article/Supplementary Materials. Further inquiries can be directed to the corresponding author.

## Supplementary Materials

The following supporting information can be downloaded at: https://www.mdpi.com/article/10.3390/microorganisms13122742/s1, Figure S1: Schematic diagram of the S-shaped five-point composite soil sampling method. Black dots (●) represent the five subsampling points arranged along an S-shaped transect to ensure representative spatial coverage of the plot. At each point, soil was collected from a depth of 0–20 cm and subsequently combined into a single composite sample for laboratory analyses; Figure S2: Differential microbial genera identified by LEfSe analysis in soils from vegetable–earthworm co-cultivation (VE) and vegetable monoculture (CK) systems under different fertilization regimes (LDA threshold = 2; a, bacteria; b, fungi); Figure S3: Structural equation models (SEMs) illustrating the direct and indirect effects of fertilization, soil properties, and microbial attributes on crop yield in soils from vegetable–earthworm co-cultivation (VE) and vegetable monoculture (CK) systems (red lines represent negative correlation, green lines represent positive correlation). Numbers along the arrows represent standardized path coefficients, indicating the strength and direction of direct effects between variables. Green and red arrows denote positive and negative relationships, respectively, and arrow thickness corresponds to the magnitude of the effect. Dashed lines indicate non-significant paths (*p* > 0.05). The goodness of model fit was evaluated using *χ*^2^/*df* (chi-square per degree of freedom), P (probability level), GFI (Goodness of Fit Index), and RMSEA (Root Mean Square Error of Approximation), where *χ*^2^/*df* < 3, *p* > 0.05, GFI > 0.90, and RMSEA < 1 indicate an acceptable model fit; Table S1: Sequence counts for 16S and ITS amplicons in each sample across the six treatments (*n* = 18); Table S2: Explained variance and significance of RDA axes for bacterial and fungal community structures at the genus level in relation to environmental factors.
