# Supplementary material for: Organic Fertilizer Substitution Modulates Soil Properties and Microbial Communities in a Vegetable–Earthworm Co-Cultivation System
Source: Microorganisms. 2025 Dec 1;13(12):2742. doi: 10.3390/microorganisms13122742 (PMC12735696; doi:10.3390/microorganisms13122742)
Supplement: Supplementary file 1 [file microorganisms-13-02742-s001.zip › microorganisms-3996662 - fc done.pdf]

microorganisms

Supplementary materials

Organic Fertilizer Substitution Modulates Soil Properties and Microbial Communities in a Vegetable–Earthworm Co-cultivation System

Shumei Cai [1,2,3], Sixin Xu [1,2,3], Deshan Zhang [1,2,3], Yun Liang [1,2,3], Xianqing Zheng [1,2,3] and Haitao Zhu [1,2,3]*

[1] Institute of Eco-Environment and Plant Protection, Shanghai Academy of Agricultural Sciences, Shanghai 201403, China; caishumei@saas.sh.cn (S.C.); xusixin@saas.sh.cn (S.X.); zds234@163.com (D.Z.); liangyun@saas.sh.cn (Y.L.); zxqfxf@163.com (X.Z.)
[2] Key Laboratory of Low-Carbon Green Agriculture, Ministry of Agriculture and Rural Affairs, Shanghai 201403, China
[3] Shanghai Key Laboratory of Horticultural Technology, Shanghai 201403, China
* Correspondence: htzhu123@163.com

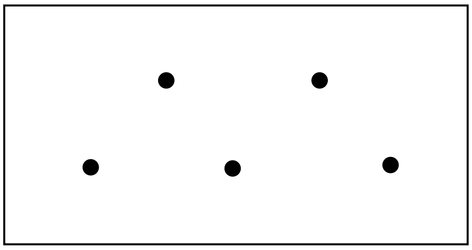

Figure S1. Schematic diagram of the S-shaped five-point composite soil sampling method. Black dots (●) represent the five subsampling points arranged along an S-shaped transect to ensure representative spatial coverage of the plot. At each point, soil was collected from a depth of 0–20 cm and subsequently combined into a single composite sample for laboratory analyses.

 https://doi.org/10.3390/microorganisms13122742

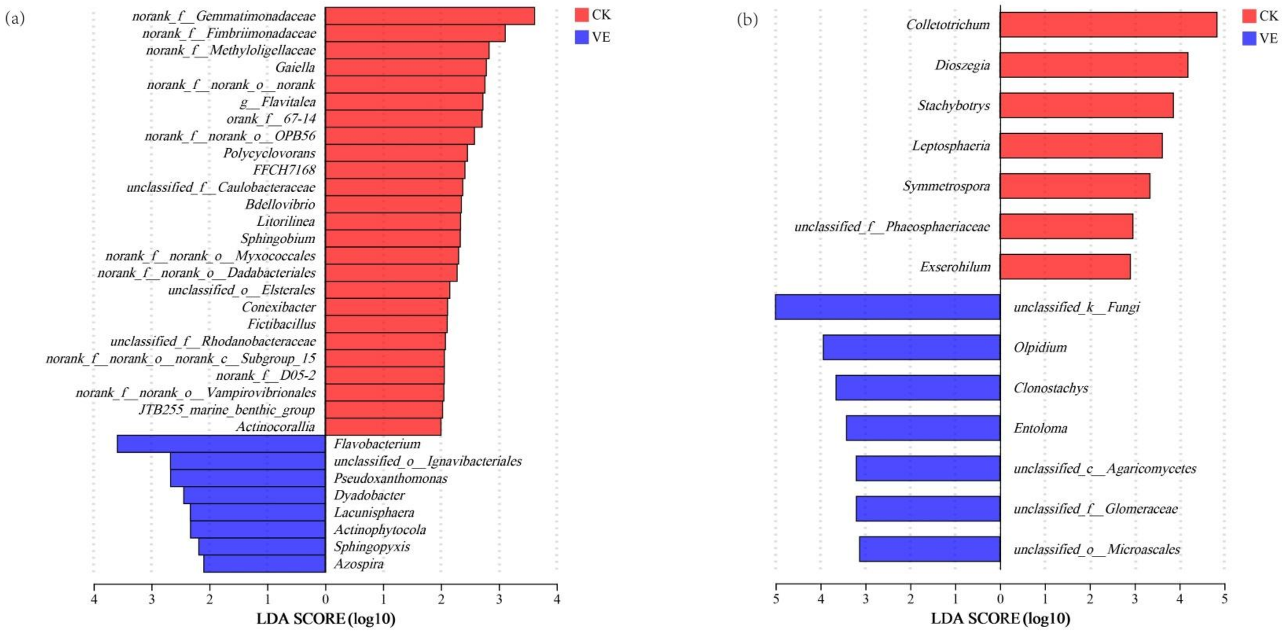

Figure S2. Differential microbial genera identified by LEfSe analysis in soils from vegetable–earthworm co-cultivation (VE) and vegetable monoculture (CK) systems under different fertilization regimes (LDA threshold = 2; a, bacteria; b, fungi).

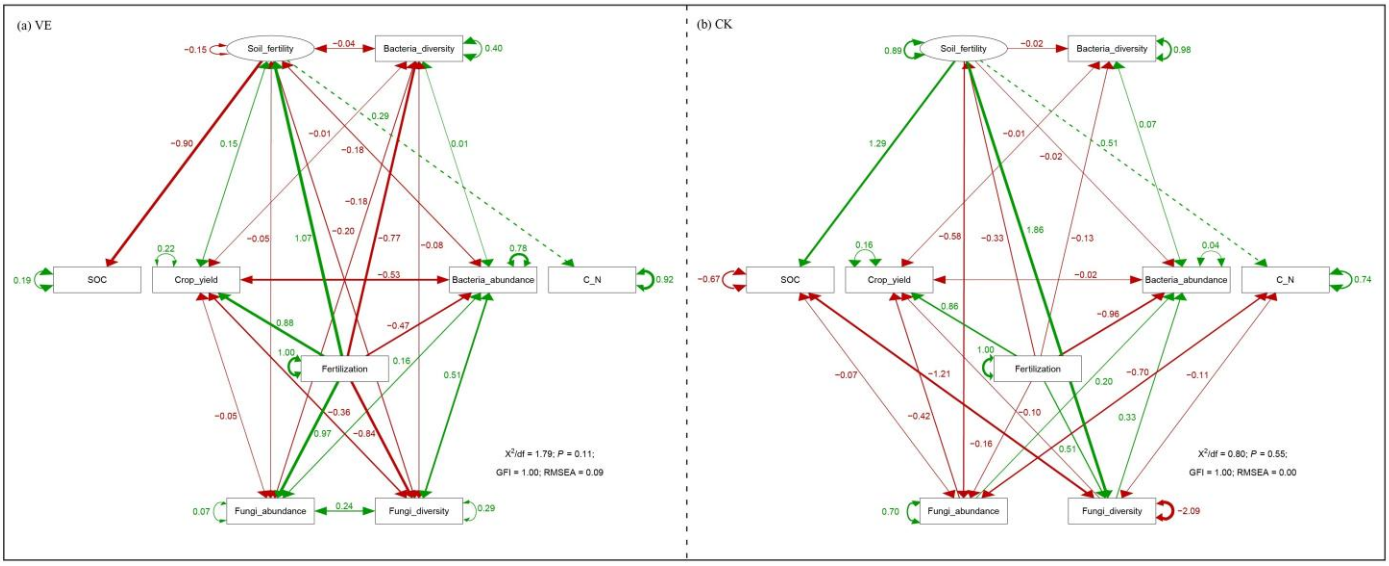

Figure S3. Structural equation models (SEMs) illustrating the direct and indirect effects of fertilization, soil properties, and microbial attributes on crop yield in soils from vegetable–earthworm co-cultivation (VE) and vegetable monoculture (CK) systems (red lines represent negative correlation, green lines represent positive correlation). Numbers along the arrows represent standardized path coefficients, indicating the strength and direction of direct effects between variables. Green and red arrows denote positive and negative relationships, respectively, and arrow thickness corresponds to the magnitude of the effect. Dashed lines indicate non-significant paths ($p > 0.05$). The goodness of model fit was evaluated using χ^2/df (chi-square per degree of freedom), p (probability level), GFI (Goodness of Fit Index), and RMSEA (Root Mean Square Error of Approximation), where $\chi^2/df < 3$, $p > 0.05$, GFI > 0.90, and RMSEA < 1 indicate an acceptable model fit.

Table S1. Sequence counts for 16S and ITS amplicons in each sample across the six treatments (*n* = 18)

Sample ID	Treatment	16S Sequence Counts	ITS Sequence Counts
CK_0_1	CK_0	43472	62345
CK_0_2	CK_0	53876	77056
CK_0_3	CK_0	49187	70752
VE_IF100_1	VE_IF100	59469	53853
VE_IF100_2	VE_IF100	70540	58205
VE_IF100_3	VE_IF100	59785	51133
VE_OF100_1	VE_OF100	47267	48129
VE_OF100_2	VE_OF100	59084	55572
VE_OF100_3	VE_OF100	54787	45152
VE_0_1	VE_0	51682	45269
VE_0_2	VE_0	58011	53938
VE_0_3	VE_0	48518	45269
VE_IF70_1	VE_IF70	46918	67891
VE_IF70_2	VE_IF70	51183	74819
VE_IF70_3	VE_IF70	44075	65120
CK_IF100_1	CK_IF100	44653	59974
CK_IF100_2	CK_IF100	48298	71430
CK_IF100_3	CK_IF100	43741	70756
**Total**		**934546**	**1076663**
**Average**		**51919**	**59815**

VE_IF100, full-rate inorganic fertilizer; VE_IF70, 30 percent reduced inorganic nitrogen fertilizer compensated by organic fertilizer; VE_OF100, organic fertilizer only; VE_0, no fertilization; CK_IF100, full-rate inorganic fertilizer without earthworms; CK_0, unfertilized control without earthworms.

Table S2. Explained variance and significance of RDA axes for bacterial and fungal community structures at the genus level in relation to environmental factors

Envfit_Bacterial community	RDA1	RDA2	R^2	*p*_value
**AP**	0.57764	-0.81629	0.70820	0.15972
**AK**	0.98569	-0.16856	0.63390	0.22639
**pH**	0.99837	0.05711	0.04400	0.94583
**AHN**	0.02571	0.99967	0.71387	0.15556
**C_N**	-0.98778	0.15583	0.98727	0.00972
**Envfit_Fungal community**	**RDA1**	**RDA2**	**R^2**	***p*_value**
**AP**	-0.83261	-0.55387	0.59274	0.18889
**AK**	-0.71777	-0.69628	0.59735	0.31667
**pH**	0.44826	-0.89390	0.71138	0.07222
**AHN**	0.58803	0.80884	0.19342	0.67639
**C_N**	0.53373	0.84565	0.90552	0.06389
